# Draft Genome Sequence of the Wild Edible Mushroom Pleurotus ostreatoroseus DPUA 1720

**DOI:** 10.1128/MRA.00840-20

**Published:** 2021-01-28

**Authors:** Suelen Dias da Silva, Eliane Carvalho dos Santos, Waldeyr Mendes Cordeiro da Silva, Alessandra Alves da Silva Magalhães, Elliza Emily Perrone Barbosa, Maria Francisca Simas Teixeira, José Odair Pereira, Adolfo José da Mota

**Affiliations:** a Biodegradation Laboratory, Bionorte Biotechnology Graduate Network, Federal University of Amazonas (UFAM), Manaus, Amazonas, Brazil; b Jungle War Instruction Center (CIGS), Brazilian Army, Manaus, Amazonas, Brazil; c NEPBIO, Federal Institute of Goiás, Formosa, Goiás, Brazil; d Department of Cell Biology, University of Brasília, Campus Darcy Ribeiro, Brasília, Distrito Federal, Brazil; e Department of Physiological Sciences, Institute of Biological Sciences, Federal University of Amazonas (UFAM), Manaus, Amazonas, Brazil; f Culture Collection DPUA, Federal University of Amazonas, Manaus, Amazonas, Brazil; Vanderbilt University

## Abstract

The draft whole-genome sequence of the mushroom Pleurotus ostreatoroseus DPUA 1720 (38,588,587 bp) is presented here. This report contributes to the prospective research for bioactive compounds in the genus *Pleurotus*.

## ANNOUNCEMENT

Pleurotus ostreatoroseus Singer 1961 is autochthonous to tropical and subtropical areas (1) with a remarkable capacity to degrade a variety of lignocellulosic residues (2). This mushroom is a potential source of bioactive compounds, including phenolic acids, organic acids, polysaccharides, and tocopherols (3–5).

**FIG 1:**
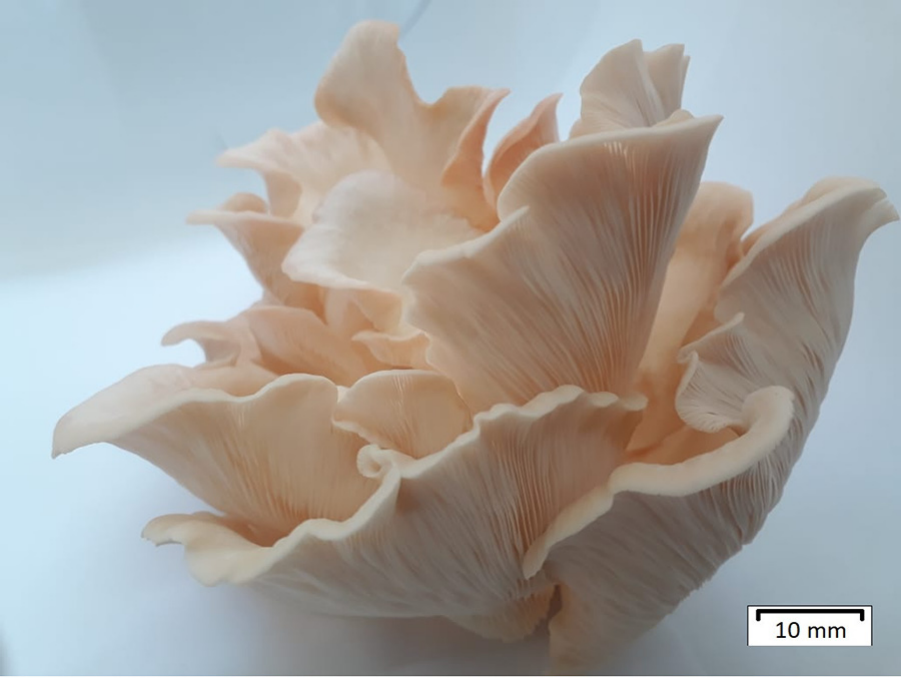
Fruiting body of *Pleurotus ostreatoroseus.*

*P. ostreatoroseus* DPUA 1720 (Fig. 1) was accessed from the Culture Collection of the Parasitology Department of Amazonas Federal University (DPUA). The mycelial mass was produced in potato dextrose (PD) broth, cultured for 72 h at 28°C with continuous shaking at 150 rpm.

Genomic DNA was prepared from the dikaryotic mycelium with an E.Z.N.A. fungal DNA minikit (Omega Biotech). The internal transcribed spacer (ITS) regions and partial sequences of 28S rRNA genes were amplified using ITS1 (5′-TCCGTAGGTGAACCTGCGG) and Uni-R (5′-GGTCCGTGT TTCAAGACG) primers (6, 7). The amplicons were sequenced using BigDye Terminator v3.1 (Applied Biosystems). DNA sequences were determined in an ABI 3500 genetic analyzer (Applied Biosystems) and confirmed by a BLASTN (8) search (GenBank accession number MH915573.1).

Short-insert genomic DNA libraries (2 × 150-bp paired-end reads) were prepared using the NEBNext Ultra II DNA library kit for Illumina (New England Biolabs), according to the manufacturer’s recommendations, and sequenced in an Illumina NovaSeq 6000 system from GenOne Soluções em Biotecnologia (Rio de Janeiro, Brazil). Illumina PCR adapters and paired-end reads were quality trimmed (Q ≤ 38) using fastp v0.20 (9). Then, 16,251 Mbp (91.37%) of filtered paired-end reads were assembled using SOAPdenovo2 v2.04 software (10). Different k-mers (35, 47, 59, 71, 83, 95, 107, and 119 by default) and adjusted parameters (“-R” to solve tiny repeats, “-F” for intrascaffold gap closure, and “-d 1” to remove k-mers with a frequency no larger than 1) were employed, looking for the maximum *N*_50_ value. The final assembly was achieved utilizing a 95-mer and those adjusted parameters. The draft genome sequence has 619 contigs spanning 38,588,587 bp (largest contig, 1,071,624 bp; smallest contig, 245 bp). The *N*_50_ value was 180,995 bp, the *L*_50_ value was 62 bp, the GC content was 52.6%, and the repetition rate was 4.67% (1,802,067 bp), as predicted by the Funannotate v1.6.0 software package (11), using RepeatModeler v1.0.11 and RepeatMasker v4.0.7.

Gene prediction was carried out using AUGUSTUS v3.2.1 (12) combined with OrthoDB v9 (13) data sets, BUSCO v3 (14), and a collection of 798,104 fungus transcript sequences from the NCBI nucleotide database, obtained by filtering for fungi/basidiomycetes/mRNAs. From the 11,296 predicted proteins available in NCBI, 1,645 were annotated. The UniFIRE workflow (15) with default parameters was implemented to identify functional annotations and dbCAN2 (16) to detect carbohydrate-active enzymes (CAZy). The dbCAN2 parameters were as follows: for HMMER, E value <1e-15 and coverage >0.35; for DIAMOND, E value <1e-102; for Hotpep, frequency >2.6 and hits >6; and for SignalP, default parameters. According to the CAZy classification (17), there were 80 auxiliary activities (AAs), 17 carbohydrate esterases (CEs), 10 polysaccharide lyases (PLs), 61 glycosyl transferases (GTs), and 158 glycoside hydrolases (GHs). A summarized annotation file containing the protein NCBI identification, the recommended name, comments about similarity, Enzyme Commission (EC) number, and CAZy results are available on GitHub (https://github.com/waldeyr/P_ostreatoroseus_DPUA_1720.git).

### Data availability.

The draft genome sequence of *Pleurotus ostreatoroseus* has been deposited in DDBJ/ENA/GenBank under accession number SWBT00000000. The raw data have been deposited in the NCBI/SRA database under accession number SRR12316382.
